# Influence of photothermal and plasma-mediated nano-processes on fluence thresholds for ultrafast laser-induced cavitation around gold nanoparticles†

**DOI:** 10.1039/d3na00743j

**Published:** 2023-10-20

**Authors:** Leonidas Agiotis, Vi Tching De Lille, Michel Meunier

**Affiliations:** a Department of Engineering Physics, Polytechnique Montréal Montreal QC H3C 3A7 Canada michel.meunier@polymtl.ca

## Abstract

Laser fluence thresholds of ultrafast excitation of vapor bubbles around gold nanoparticles are determined experimentally. An optical scattering technique of limited minimum bubble size resolution is employed and analyzed for that purpose. Measurements were performed for spherical gold nanoparticles of varying sizes (40–200 nm) and for laser pulses of varying pulse width (55 fs to 4.3 ps) to estimate the limits where the evaluated thresholds are attributed to either plasma-mediated or photothermal cavitation. Furthermore, thresholds were obtained by double 55 fs pulsed excitation (varying delay 0.0–4.3 ps), providing insights into the dynamics of the excited plasma. A relationship is established between particle properties, (size, near-field amplification factor, and absorption efficiency) and the crossover pulse width of the transition from plasma-mediated to photothermal cavitation. Further, by comparing theory and experiments, we examine the approximative optical breakdown density of ∼10^−21^ cm^−3^ at a distance of 1–2 nm from the particle surface as a criterion of plasma-mediated cavitation around gold nanoparticles in analogy to the spinodal criterion for photothermal cavitation. For a given pulse width, the breakdown density appears to be nearly size-independent, establishing the aforesaid criterion applicable. However, a small pulse width dependence of the breakdown density is still observed. Based on these criteria, a comparison is further provided between theoretical thresholds of cavitation and the ones of detectable bubbles. An increasing discrepancy is observed between them with decreasing size for the case of photothermal cavitation. For plasma-mediated cavitation, the latter discrepancy is seemingly smaller, presumably due to the highly nonlinear nature of the process.

## Abstract 2

This work builds on an in-depth evaluation of cavitation bubbles around gold nanoparticles upon ultrafast laser photoexcitation and the transition of photothermal to plasma-mediated cavitation, depending on particle properties and laser pulse width.

## Graphical abstract

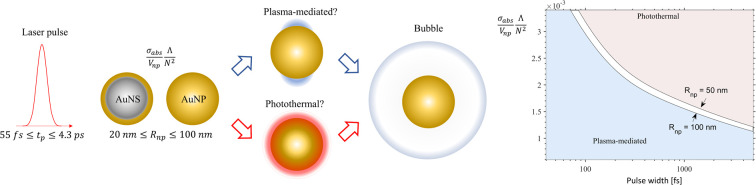

## Introduction

Recent interest has developed in the excitation of localized plasma in water adjacent to gold nanoparticles by high-intensity laser radiation.^1–7^ A straightforward relation has been established with (among other applications) the ultrafast laser-induced breakdown nano-surgery of cellular media,^8–10^ with implications of performing highly localized incisions on cells.^11^ The idea involves non-resonant and ultrafast interaction between the laser field and the excited plasmon mode of a gold nanoparticle.^12^ Thus, even though energy is not efficiently stored in the plasmon mode itself, high-contrast localized nonlinear ionization is achieved in the aqueous medium near the particle because of broadband plasmon-induced near-field amplification. A distinction between this type of *plasma-mediated* bubbles and the *photo-thermal* bubbles induced by absorption and heating of gold nanoparticles is therefore of critical importance to applications, which in turn requires a clear determination of their respective threshold laser fluences.

In the case of photothermal vapor bubbles the fluence thresholds have been determined over the years by various experimental techniques^13–17^ and compared with theoretical simulations.^18–20^ Experimentally, the reported thresholds are governed by the limits of detection of various employed techniques: the most sensitive hitherto being the X-ray scattering^15,21^ and the transient extinction pump–probe^13^ techniques, with reported minimum detected bubble diameters of ∼10 nm^15,21^ and ∼40 mn,^13^ respectively. An optical scattering technique, as reported by Lapotko,^22^ allowed the detection of bubbles with a minimum lifetime of ∼15 ns (implying a minimum bubble diameter of ∼160 nm, accounting for the Rayleigh–Plesset relationship *τ* ≈ 0.092*d*_max_). From a theoretical point of view, Lombard *et al.*,^18^ by comparison of experimental thresholds^13–15^ with calculations based on a free energy hydrodynamic model and a simplified thermal model, have recently proposed the following bubbling criterion: laser-induced photothermal bubbles around a spherical gold nanoparticle are excited as long as the spinodal temperature of water is crossed at a distance between 1–2 nm (for nanoparticles of sizes 5–200 nm).

For plasma-mediated nano-cavitation, a theoretical model has been recently developed^23^ based on data obtained by a shadowgraphic imaging technique^24^ and an optical scattering technique.^25^ Notably, the shadowgraphic imaging technique allows for minimum detectable bubble diameters of ∼0.8–1.0 μm. Theoretical investigations^23^ were accordingly based on the threshold fluence of detectable vapor bubbles. It was shown that the total energy density deposited around a thin layer of water around a single gold nanoparticle determines the threshold fluence of cavitation, independent of the channel of energy deposition, *i.e.*, by thermal conduction at the particle interface or by plasma relaxation.

The foregoing discussion raises several questions related to the plasma-mediated nano-cavitation thresholds around gold nanoparticles. To begin with, can the detection sensitivity of the employed techniques be improved? Of particular interest is the optical scattering technique owing to its simplicity and practicality over the more sensitive, yet complex, pump–probe spectroscopy or X-ray scattering techniques. Indeed, the optical scattering technique can potentially reach higher detection resolution compared to shadow graphic imaging, however, the development of a systematic formalism is required: for instance, the probability detection threshold has been determined by the 50% probability,^26,27^ which varies with particle concentration. In addition, the spinodal bubbling criterion proposed by Lombard *et al.*^18^ to predict photothermal bubbles, raises the question of whether an analogous criterion can be employed for the case of plasma-mediated bubbles (*i.e.*, the approximative optical breakdown density of ∼10^−21^ cm^−3^ is reached at a thin layer of 1–2 nm from the particle surface). Such criterion would be particularly useful for practical applications to predict either photothermal or plasma-mediated cavitation at favored laser fluence. Finally, it would be critical to examine the influence of plasma dynamics within the fs to ps range at the cavitation threshold, especially for decreasing particle sizes (<80 nm), where electron diffusion is predicted to affect substantially plasma localization.

In this work, we provide an analysis of the optical scattering technique for nanobubble detection around gold nanoparticles. Our analysis allows systematic determination of the fluence threshold of nanobubbles of a specific size (∼400–500 nm) determined by the detection limit of the apparatus, *i.e.*, higher than the very fluence thresholds of cavitation. Accordingly, we experimentally measure the threshold fluence of detectable vapor bubbles around gold nanoparticles of varying sizes (40–200 nm) excited by varying ultrafast laser pulse widths (55 fs to 4.3 ps). The aforesaid threshold fluence is further determined by double 55 fs pulses of varying delays (0–4 ps) to investigate the influence of plasma relaxation in the process. Based on all experimental observations we employ simplified numerical models to evaluate the involved processes at the cavitation thresholds (photothermal or plasma-mediated). We finally aim to examine whether a bubbling criterion can be determined in the case of plasma-mediated cavitation, in analogy to the spinodal criterion proposed by Lombard *et al.*^18^ for the case of photothermal nanobubbles. That will allow us to provide a discussion on observed discrepancies between the thresholds for detectable bubbles by the optical scattering technique and theoretical thresholds.

## Methods

**Fig. 1:**
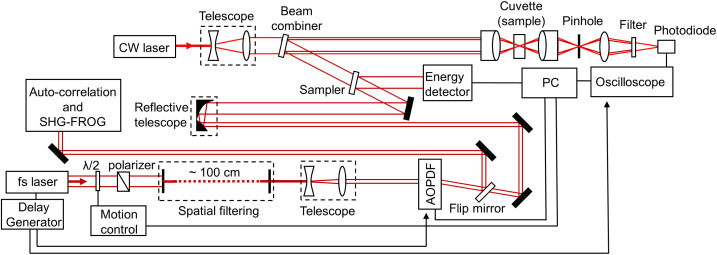
Graphical representation of the experimental setup (see text for details).

The technique used in this work has been previously employed for the detection of nanobubbles.^12,14–16,22,25–27^ A schematic of the experimental setup is shown in Fig. 1. A femtosecond laser amplifier generates pulses of ∼70 fs (FWHM) with a central wavelength of 800 nm, bandwidth of ∼25 nm, and energy of ∼3.4 mJ. At the optimum compressor configuration of the laser system, the pulses were found to possess reminiscent dispersion (evaluated by a second harmonic generation FROG technique). The pulse energy was controlled by a combination of a half-waveplate and a polarizer. The nominal ∼10 mm 1/*e*^2^ beam diameter at the output of the amplifier was subsequently “cleaned” by a combination of two irises. The first iris was set at an aperture diameter of ∼1.8 mm, and the second iris was positioned ∼100 cm apart, partially open so that high-order diffraction is filtered out. The transmitted Airy spot was magnified to a 1/*e*^2^ radius ∼1.15 mm by a telescope to fill the entrance window of an acousto-optic programmable dispersive filter (AOPDF, Dazzler, Fastlite). The latter was employed for pulse dispersion control, pulse measurements, pulse stretching, and double pulse generation. A flip mirror was positioned at the output of the AOPDF to send the pulses for temporal characterization. The estimated reminiscent dispersion at the output of the amplifier (as determined by FROG measurements), was introduced in the AOPDF and thus was compensated. As a result, the pulses were further compressed down to a minimum of ∼55 fs. Second-order dispersion was then introduced by the AOPDF to stretch the pulses up to ∼4.3 ps, as measured by scanning intensity autocorrelation. When the flip mirror was cleared out, the beam was sent to the far field to a 3× magnifying reflective telescope, allowing better focusing into the samples. The beam was sampled toward an energy detector and then combined with a CW He–Ne laser source (used as a probe beam), of which the 1/*e*^2^ radius has been expanded to ∼5.9 mm. The two beams were focused collinearly by a 0.17 NA microscope objective and characterized near the focus by a knife-edge technique (pump beam focal 1/*e*^2^ radius ∼7.3 μm and Rayleigh length ∼110 μm, probe beam focal 1/*e*^2^ radius ∼3.3 μm and Rayleigh length ∼20 μm (ESI, Fig. S1a†)). A 10 mm thick quartz optical cuvette was used to hold the samples. For all experiments, the cuvette entrance was placed ∼1 mm in front of the focus, to minimize absorptive losses. A second microscope objective collected the transmitted light and imaged it through a ∼10 μm pinhole. The pump beam was finally filtered out, while the probe signal was collected by a 2 GHz (SV2-FC, Thorlabs) photodiode and was fed to an oscilloscope. The repetition rate of the laser system was set to 50 Hz. The power-dependent signal collection was automatized around the detectable cavitation thresholds. At each input laser energy (fluence), 20 scope signals were collected and subsequently normalized to the background noise. A moving average and a lowpass filter at 5 MHz removed the noise. The detection probability was finally calculated by registering signals that were equal to five times the noise standard deviation. Bubble signals of minimum lifetimes as low as ∼40–50 ns were recorded by the system. Thus, we estimate detectable laser-induced bubbles of minimum size ∼0.4–0.5 μm when the nanoparticle was subjected at a fluence superior to a threshold value within the imaged volume of the probe beam.

As for the examined samples, aqueous colloidal solutions of Au nanoparticles were purchased from NanoComposix and NanoPartz. Bare (citrate) spherical gold nanoparticles (AuNP) of 40, 60, 80, and 100 nm (NanoXact, NanoComposix), 150 and 200 nm (NanoPartz) diameter and gold nano-shells (AuNS) with PEG ligand (5 kDa), ∼80 nm inner silica diameter, ∼20 nm gold shell thickness, ∼120 nm outer diameter and surface plasmon resonance (SPR) peak ∼660 nm (NanoXact, NanoComposix) were selected for our studies. The solution concentrations were adjusted by diluting the chosen portion of stock colloidal solution (of determined nominal concentration) with distilled water.

A statistical relation between the detection probability of a bubble and the detection effective volume can be formulated by defining the parameters: imaged effective volume *V*^i^_eff_, the volume of a single nanoparticle *v*_np_ and the concentration *c*. Following other approaches, which have been employed for the laser-induced breakdown spectroscopy technique,^28–31^ we define the single trial probability of success *p* = *cv*_np_ of detecting *k* bubbles around *X* nanoparticles for a given number *n* = *V*^i^_eff_/*v*_np_ of possible displacements within the volume *V*^i^_eff_. The binomial mass function of the detection probability then reads:^29,31^
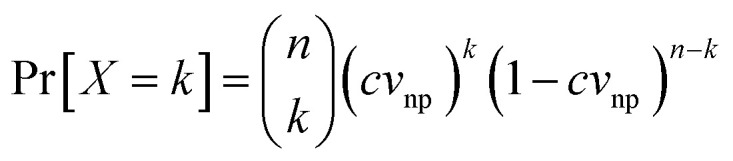
In our configuration, it holds typically *v*_np_ ≪ *V*^i^_eff_. Thus, since the single trial probability *p* → 0 while *n* → ∞, we can invoke the Poisson mass function Pr[*X* = *k*] = e^−*μ*^*μ*^*k*^/*k*! where the variable *μ* = *cV*^i^_eff_ expresses the mean number of bubbles detected within the volume of interest. Finally, the cumulative distribution function of having at least one bubble excited within the volume of interest reads:^32^1
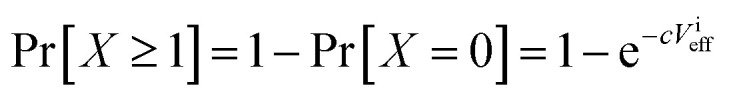


It remains to determine the dependency of the cumulative probability function on the input laser fluence. The laser fluence distribution of the pump beam is assumed Gaussian so that 
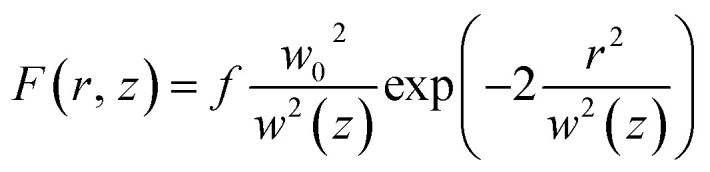
 where *f* denotes the peak fluence, *w*(*z*) is the 1/*e*^2^ beam radius and *w*_0_ the minimum radius at the focus, connected by the relation 
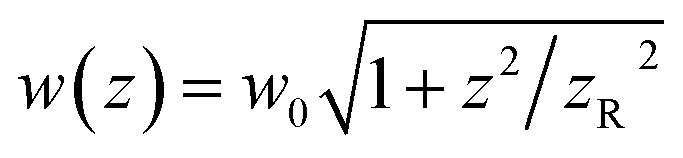
, with *z*_R_ denoting the Rayleigh length. Thus, we can express the effective volume of the pump laser *V*_eff_(*f*), as a function of peak fluence *f* by 
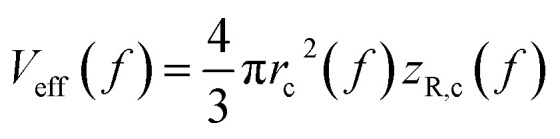
, where *r*_c_(*f*) and *z*_R,c_(*f*) define the transverse and longitudinal distances of the ellipsoid where *F*(*r*, *z*) = *f*. Consequently, everywhere within the enclosed volume *V*_eff_(*f* > *F*_t_), the peak fluence *f* surpasses the threshold fluence *F*_t_. Following,^29,31^*r*_c_(*f*) and *z*_R,c_(*f*) can be related to *F*_t_ by setting *F*(*r* = *r*_c_, 0), *F*(0, *z*_R_ = *z*_R,c_), respectively, so that we can finally express the effective volume as:
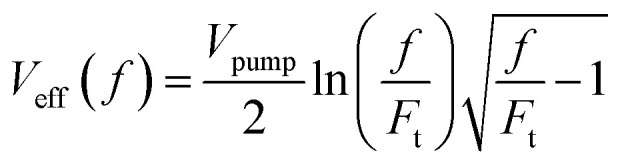
where 
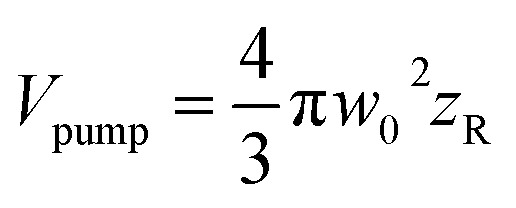
. The values *w*_0_ and *z*_R_ have been determined experimentally within the paraxial Gaussian beam propagation relations.

The effective volume has been expressed by considering how the peak fluence of the pump beam increases. Nonetheless, the signal related to cavitation detection originates from a volume related to the depth of focus of the probe beam, defined as 
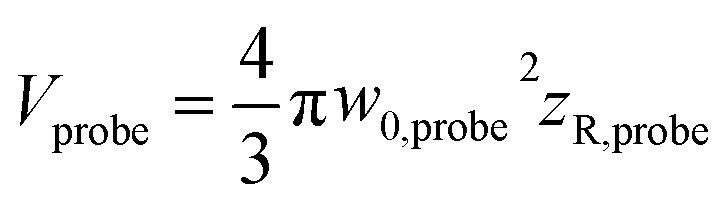
 (ESI, Fig. S1b†). In fact, *V*_eff_(*f* ≈ 1.1*F*_t_) = *V*_probe_, which shows that for *f* > 1.1*F*_t_ bubbles will be excited outside the volume *V*_probe_. Since pump and probe beam fluence profiles share the same functional form, we can assume that the probe beam images a portion ∼*V*_probe_/*V*_pump_ of the effective volume *V*_eff_(*f*). Thus, the imaged effective volume *V*^i^_eff_ can approximately be expressed by2
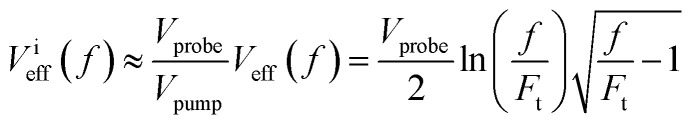


Based on the described probability formalism, nanoparticle solutions of varying concentrations were tested. The fluence threshold was determined for AuNP samples by varying their concentration. The cumulative probability function was fitted on the retrieved data set for the corresponding *μ* values, as defined by the applied concentration values and their absolute uncertainties. A nonlinear least squares method was employed (by use of the curve fitting toolbox of MATLAB) to fit eqn (1) and (2) on the retrieved data with the threshold fluence as the fitting coefficient for a known concentration. The process yields the threshold fluence value with its 95% confidence bounds. To determine the absolute uncertainties of the determined threshold fluence, the following methodology was applied: first, the upper bound of concentration (determined by the absolute uncertainty) was applied to the fitting of eqn (1) and (2) to measured data. The upper 95% confidence bound of the fitting coefficient (fluence threshold) then defined the upper absolute uncertainty of the fluence threshold measurement. Subsequently, the process was repeated for the lower bounds.

**Fig. 2:**
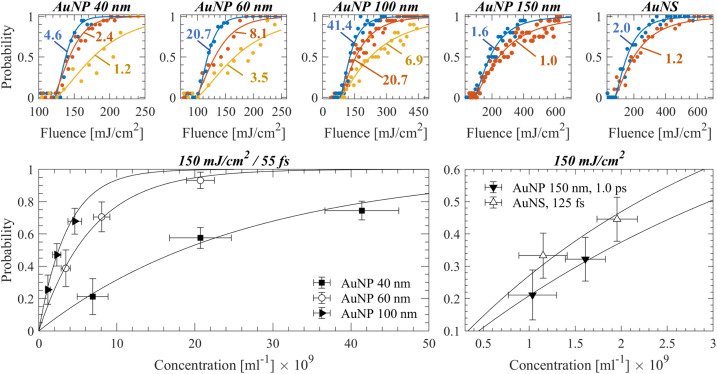
Top panels: Detection probability curves obtained experimentally for a specific nanoparticle sample/irradiation regime, at varying concentrations. Circles indicate experimental measurements and lines, fitting curves based on eqn (1) and (2). The examined concentrations in each figure (shown in 10^9^ ml^−1^) correspond to the ones indicated in the figures on the bottom panel. As the concentration becomes smaller, the fluence that corresponds to a 50% detection probability increases. Bottom panels: Detection probability as a function of particle concentration is plotted for the experimental data, which demonstrates a good agreement with the proposed model of eqn (1) (shown with solid black lines).

In Fig. 2, detection probability measurements of various samples are shown. For each sample, the determined threshold fluences were averaged and used as inputs for estimating the probability dependence on particle concentration at an excitation fluence. The absolute probability uncertainties were estimated by the mean square error of eqn (1) and (2) fits. A good agreement is observed between the proposed model and the experimental measurements for various conditions.

The uncertainty in the calculation of the fluence thresholds includes further the absolute uncertainty of laser power variation. Larger dispersion values or pulse delays introduced in the acousto-optic programmable dispersive filter resulted typically in higher shot-to-shot laser power fluctuations. Moreover, it becomes evident that the 50% probability varies significantly as a function of concentration among samples, yet such parameters as the pulse width and particle size/type uniquely correspond to a threshold fluence value.

## Results

**Fig. 3:**
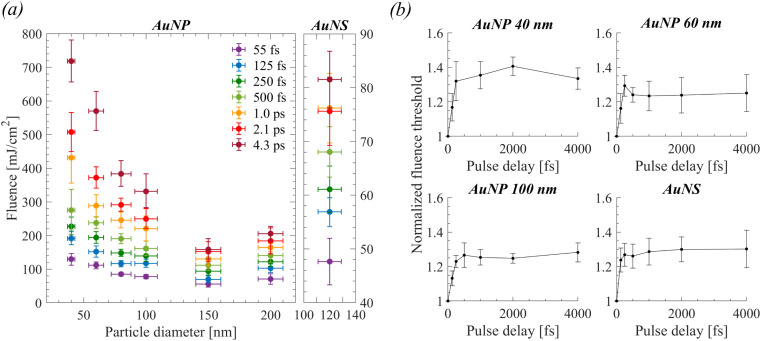
(a) Size-dependent, experimentally evaluated fluence thresholds of detectable cavitation bubbles of spherical AuNPs (left) and the ones of AuNS (right) for all applied laser pulse widths. (b) Results of double 55 fs pulse experiments as a function of pulse delay. All fluence thresholds have been normalized to the one acquired under single 55 fs pulse excitation.

Fig. 3a shows the particle size and pulse width dependence of the fluence thresholds determined experimentally. Qualitatively, the behavior agrees with the dependency of demonstrated data in ref. 23. The overall minimum fluence threshold is obtained for spherical particles of ∼150 nm diameter for any given pulse width. Particularly for the shortest pulses (*t*_p_ = 55 fs), this effect can be associated with the squared field amplification *N*^2^ dependency on the pump wavelength 800 nm as a function of particle size (Table 1), which in turn implies plasma-mediated cavitation (shown experimentally in our previous work^25^). The field amplification is defined as *N* = *E*_1_/*E*_in_, where *E*_1_ denotes the local amplified field near the particle and *E*_in_ the input field of the laser light. Indeed, since *I*_in_ ∝ |*E*_in_|^2^, by assuming a critical local intensity for a plasma-mediated cavitation process as *I*_c_ ∝ |*E*_1_|^2^, then *I*_c_ = *N*^2^*I*_in_ ∝ *N*^2^*F*_th_/*t*_p_. Therefore, it is estimated that the minimum measured threshold fluence is obtained for the particle of maximum *N*^2^ (*i.e.*, for 150 nm particles). Further, for *t*_p_ = 55 fs, the fluence threshold for any given particle can be related to the minimum fluence threshold by a factor *N*_max_^2^/*N*^2^ with a satisfactory agreement (ESI, Section S2c†), which corroborates the hypothesis for plasma-mediated cavitation at this pulse width. In addition, calculations based on a thermal model alone cannot explain a photothermal cavitation process in that case (ESI, Section S2c†). Notably, the relation of fluence threshold on pulse width resembles the plasma-mediated cavitation in pure water.^10^ Longer employed pulse widths require monotonically increasing fluence so that a bubble of the same size is detected. By increasing particle size, the relative amount of excess energy required for bubble detection at longer pulse widths compared to shortest ones decreases and becomes minimum for 150 nm particles. The aforementioned excess energy becomes even smaller for the case of AuNS, for which the pulse width-dependent fluence thresholds are also shown in Fig. 3a. In that case, the fluence threshold is leveling off for pulse widths longer than 500 fs around ∼75–80 mJ cm^−2^. Accordingly, there are strong implications for photothermal bubbles at longer pulse widths while the volumetric absorption of the particles *σ*_abs_/*V*_np_ (calculated by Mie theory) becomes considerably higher (Table 1).

**Table 1:** Calculations performed based on Mie theory to determine the squared near-field amplification *N*^2^ at 800 nm (at the surface of the particle, where it becomes maximum, and at a radial distance *r* = 1.5 nm away from it), the characteristic diffusion length *Λ* for the examined nanostructures, (taken as the distance at which *N* is reduced by one half of its maximum value), the absorption cross-section *σ*_abs_ and the volumetric absorption *σ*_abs_/*V*_np_

Sample	*N* ^2^ (*r* = 0 nm), (*λ* = 800 nm)	*N* ^2^ (*r* = 1.5 nm), (*λ* = 800 nm)	*Λ* [nm]	*σ*_abs_ [nm^2^], (*λ* = 800 nm)	*σ*_abs_/*V*_np_ [10^−4^ nm^−1^], (*λ* = 800 nm)
AuNP 40 nm	13.47	10.04	9.7	22.8	6.8
AuNP 60 nm	14.98	12.18	14	89	7.8
AuNP 80 nm	17.22	14.75	19	248	9.3
AuNP 100 nm	20.25	17.81	22.6	577	11
AuNP 150 nm^a^	26.73	24.2	32	2560	15
AuNP 200 nm^a^	19.54	18.15	43	3980	9.5
AuNS^b^	34.81	31.02	26.3	3470	38

a Because of pronounced electromagnetic retardation, the *N* was calculated axially from the center of the particle at an angle (instead of parallel) to the laser polarization.

b The influence of PEG was considered by setting a medium layer refractive index of 1.4, which brings the theoretical plasmon resonance peak to one of the samples.

A series of double (55 fs) pulse experiments were further performed to determine the threshold fluence as a function of the temporal delay between pulses (0.0–4.3 ps). The objective of these experiments was to explore the influence of plasma losses (by diffusion, recombination, or collisions) or transient effects on the ultrafast bubble cavitation process. As shown in Fig. 3b, for all examined particles the cavitation threshold increases by a factor of ∼1.3 (uncertainty ≲ 10%) after a pulse delay of 500 fs and remains almost constant for increasing delays up to ∼4 ps.

Based on the above observations, and in accordance with our previous work,^12,23,25^ for spherical Au particles, plasma-mediated cavitation is expected for a single 55 fs pulse. Therefore, the result of the double pulse experiment implies that the field amplification of the particles must remain approximately constant after the excitation by the first pulse and that the excited plasma density is maintained in the vicinity of the particle even after ∼4 ps. This suggests that plasma diffusion away from the particle must be negligible within that timeframe, even for small nanoparticles. Furthermore, most likely electron thermalization occurs at larger timescales so that the first pulse does not substantially increase the temperature of the surrounding water before the arrival of the second pulse.^10^ The results in the case of AuNS, exhibit similar behavior, therefore the observed bubbles are presumably not photothermal.

## Discussion

### Modeling of plasma-mediated cavitation and the importance of relaxation processes

It is possible to calculate the time evolution of the electron density near the poles of a nanoparticle at the measured bubble detection fluence threshold. A critical electronic density is the starting point of plasma-mediated cavitation around nanoparticles and its calculation is useful for the estimation of the relative contribution of plasma-mediated *versus* photothermal processes. Free electron density *n* at a specified location near the poles of a nanoparticle (*i.e.*, where field amplification occurs) following excitation, can be estimated by the well-known rate equation:^8,10,12,25,33^3
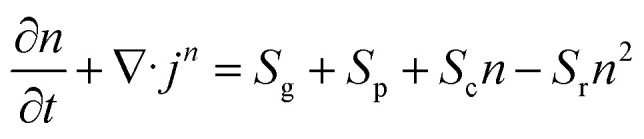
where *j*^*n*^ is the electron flux due to diffusion, *S*_g_ expresses the thermionic emission rate, *S*_p_ stands for the photoionization rate, *S*_c_ denotes the collision ionization rate and *S*_r_ is the recombination rate. The source terms *S*_p_, *S*_c_ and *S*_r_ are detailed in the ESI,† for which the local laser intensity is taken as *N*^2^*I*, where *N* is the plasmonic near-field amplification estimated based on Mie theory as a function of distance from the particle surface^34,35^ (Table 1). The gradient of the electron density flux is given by the expression ∇·*j*^*n*^ ≈ *D*_e_*n*/*Λ*^2^, where the characteristic diffusion length *Λ* is given in Table 1, *D*_e_ = *τE*_av_/(3*m*_e_) is the diffusion coefficient, *E*_av_ = 5*Δ̃*/4 is the average energy of electrons, *τ* is the time between collisions, *Δ̃* is the effective ionization potential and *m*_e_ is the electron mass.

The double pulse experiments demonstrated an excess required fluence for cavitation, which can be attributed to either electronic losses or transient absorption effects.^36^ However, as shown by pump–probe spectroscopy,^13,37^ the absorption of particles is expected to transiently (marginally) increase at the pump wavelength (800 nm), which contradicts the observation for excess fluence requirement. Thus, the effect is most likely associated with electronic losses. Even though the plasma density diminishes due to recombination or collisions, such losses contribute to water heating following thermalization. However, losses due to electron diffusion result in a dramatic decrease of plasma density at a timescale of 4 ps, unless excited electrons do not diffuse considerably away from the particle before the arrival of the next pulse. Indeed, this can be explained by the fact that, for increasing electronic densities, there is a transition from free diffusion toward the regime of ambipolar diffusion.^38–40^ The latter occurs when the Debye length 
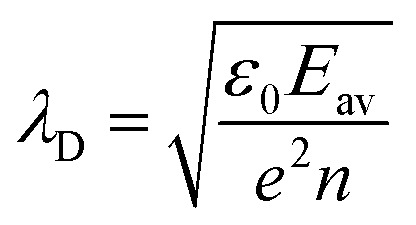
 becomes much smaller than the diffusion length, so that a space charge electric field due to the drift of charged particles can balance closely with electron diffusion. Effectively, the generated electrons sit within an induced electric field potential barrier and diffusion becomes negligible within the examined timeframes. Thus, for the electron current density term of eqn (3), advection effects due to the drift of charged (both positively and negatively) particles need to be considered.

Under the assumption of isothermal plasma, the ambipolar diffusion coefficient *D*_a_ is estimated 
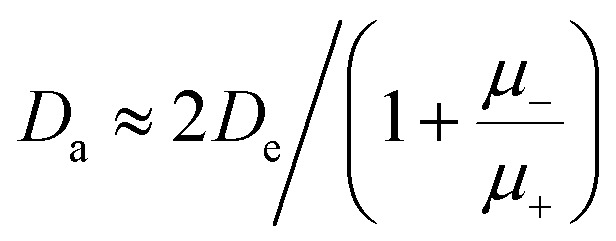
,^38^ where *μ*_+_ and *μ*_−_ stand for the mobilities of positively charged ions and electrons, respectively. When the electronic density becomes high enough so that the Debye length *λ*_D_ ≈ *Λ*, the ion density is about twice the one of the electrons, and the effective diffusion coefficient *D*_r_ is about twice the ambipolar diffusion coefficient *D*_r_ ≈ 2*D*_a_ ≈ *D*_e_/8.25 × 10^3^, considering that 
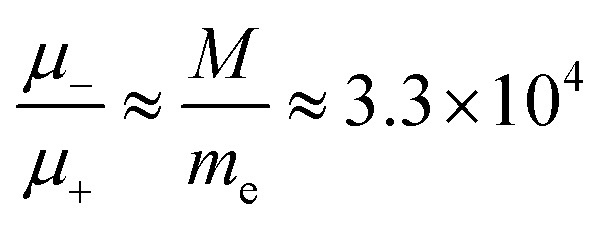
. Accordingly, the transformation *Λ* → 90*Λ* is performed in the solution of eqn (3), when *n* = *ε*_0_*Δ*/(*eΛ*)^2^ (*e.g.*, as in ref. 39).

**Fig. 4:**
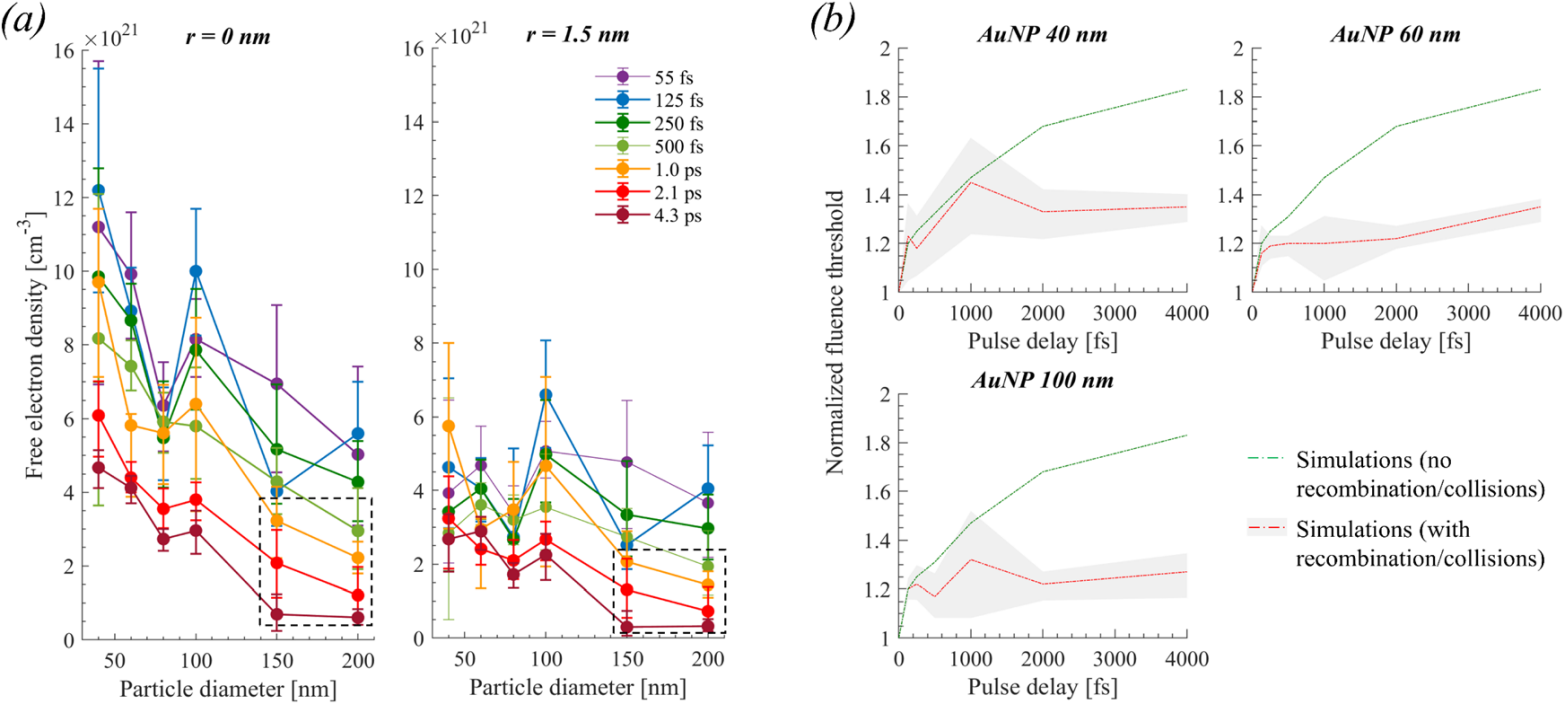
(a) Maximum (in time) electronic densities calculated at the experimental fluence thresholds, at the poles of the particles (*r* = 0 nm), and a distance (*r* = 1.5 nm) away from the pole. Dashed boxes indicate conditions where photothermal effects influence bubble formation (as demonstrated below in Fig. 5). (b) Calculation of required fluence threshold after double 55 fs pulse excitation as a function of pulse delay. The data of (a), for *r* = 0 nm, was used as a reference to estimate by numerical simulations the theoretical normalized fluencies of the double pulse experiments shown in panel (b). Particularly, in (b), for the green solid curves, we considered that under double pulse excitation, the electronic density calculated for a single 55 fs pulse must be attained at the detected thresholds. For the red solid curve (error shown by grey area) we considered that approximately the electronic densities calculated for a longer pulse (*e.g.*, 1.0 ps) must be attained at the detected threshold when the delay between double pulses increases (*e.g.*, at 1.0 ps delay), which accounts for recombination/collision effects before the arrival of the second 55 fs pulse.

In each case of nanoparticle, we can estimate the electron density produced at the poles of the particle at the determined thresholds for a single 55 fs pulse (Fig. 4a). Considering excitation by double pulses, the solution of eqn (3) shows that the excited plasma by the first pulse rapidly relaxes after excitation. Therefore, the maximum electron density excited by the second pulse decreases by increasing the pulse delay (for the double pulse of constant overall fluence). Therefore, an excess fluence is required as the delay increases to reach the same maximum electron density, as observed experimentally for the cavitation threshold. The excess fluence needed so that double 55 fs pulses attain the maximum calculated electronic densities of a single 55 fs pulse was estimated and shown in Fig. 4b (green solid curves). Notably, with increasing delay, the calculation increasingly diverges from the experimental value. Consequently, the dynamic behavior of the density of free electrons due to recombination or collisions before the arrival of the second pulse needs to be accounted for since these phenomena still participate in the heating of water following thermalization.

We can incorporate an approximate correction in our calculations by observing that the required electron densities to attain the bubble threshold are changing as a function of pulse width, as seen in Fig. 4a. For instance, for a 100 nm AuNP, an electronic density of ∼3 × 10^21^ cm^−3^ is calculated for a single pulse of ∼4.3 ps. Accordingly, we can estimate that this must be close to the threshold electron density for double 55 fs pulse excitation with a delay of ∼4 ps, *i.e.*, approximately equal to the pulse width of a single pulse excitation. Indeed, a satisfactory agreement between simulations and experiments is observed when the described methodology is applied to the calculations. Therefore, it is implied that a combination of transient effects in the plasma (most likely electron recombination and collisions) reduces the required maximum densities for the induction of a cavitation bubble when increasing the inter-pulse delay of double 55 fs pulsed excitation, much like when increasing the pulse width of single pulse excitation.

### Relative contributions of photothermal and plasma-mediated processes

**Fig. 5:**
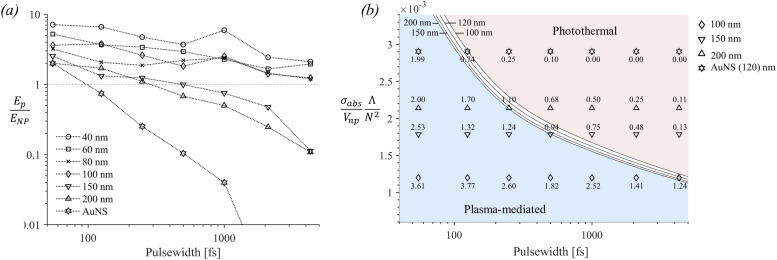
(a) Calculated ratio of the energy deposited in the plasma (*E*_p_) and in the nanoparticle (*E*_NP_) at the experimental threshold fluencies. (b) Diagram of the ratio 
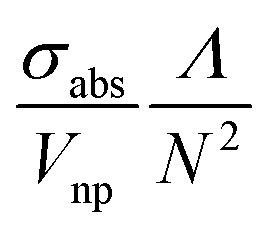
*versus* pulse width. The solid black curves correspond to the crossover pulse width *t*_p,t_, which marks the transition from plasma-mediated to photothermal cavitation for various nanoparticle sizes (shown in [nm]). The curves were calculated based on eqn (4) and numerical analysis shown in Section S3 of the ESI.† The ratios *E*_p_/*E*_NP_ for the cases of AuNS, 200 nm, 150 nm, and 100 nm particles, as shown in (a), are included for comparison. For the cases of 80 nm, 60 nm, and 40 nm particles, 
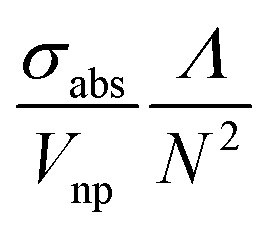
 becomes smaller and all their corresponding values reside within the plasma-mediated region, below the transition curve (not shown in the diagram).

Solutions of eqn (3) (Fig. 4a) can further be used to distinguish between plasma-mediated and photothermal cavitation at the experimental thresholds. An approximation can be obtained of the energy deposited in the plasma as 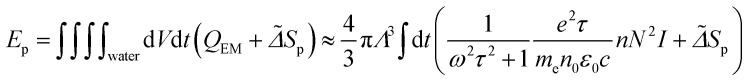 and the energy deposited at the particle as *E*_NP_ = *σ*_abs_*F* at the experimental thresholds.^12,23,25^ The calculations of the energy ratios are shown in Fig. 5a. Notably, for larger particles (150 nm and 200 nm) photothermal bubbles are implicated for pulse widths >2.0 ps, where *E*_p_/*E*_NP_ ≪ 1. The same applies for AuNS for pulse widths >250 fs, which is attributed to their higher *σ*_abs_ values. For all other particles cavitation at the experimental thresholds is seemingly dominated by a plasma-mediated process.

The determining parameters for the energy balance equation are the parameters *σ*_abs_/*V*_np_, *N*, and the diffusion length *Λ* of the particle. We can accordingly deduce an approximative relationship between them, determining the crossover pulse width *t*_p,t_ that marks the transition from plasma-mediated to photothermal cavitation process. Physically, this occurs when the amplified laser intensity in the water near a particle *I*_w_ required for obtaining a critical density (Fig. 4) is equal to the required critical intensity absorbed by the particle *I*_np_ to obtain photothermal nucleation, deposited within the corresponding interaction volumes. For photothermal nucleation, an analytical relationship has been deduced,^41,42^ reading 
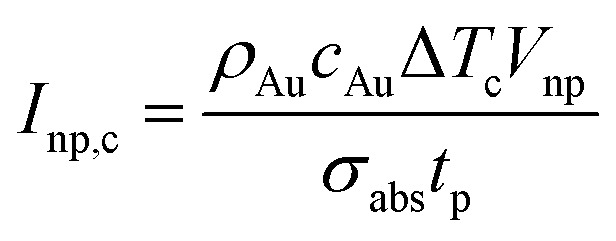
, where *ρ*_Au_ and *c*_Au_ are the density and heat capacity of gold, respectively, and Δ*T*_c_ is the critical temperature increase reached in the nanoparticle, showing that the fluence threshold is inversely proportional to the volumetric absorption of the particle under ultrafast laser excitation.^43^ Following Kennedy *et al.*,^8,44^ we can approximate the critical intensity for optical breakdown by assuming that most of the energy is absorbed by cascade ionization, so that 
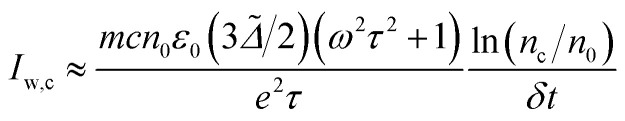
, where *n*_c_ denotes the critical electron density, *n*_0_ the seed electronic density provided by multiphoton ionization (this parameter can also include losses) and *δt* the breakdown time that *n*_c_ is reached following excitation. Therefore, at the transition, it should hold *N*^2^*I*_np,c_ ≈ *βI*_w,c_, so that:4
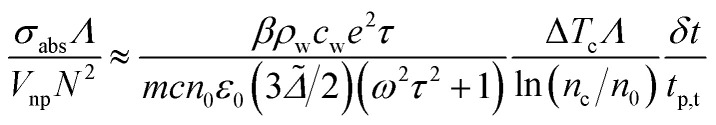
where we find *β* ≈ 3/2 for 
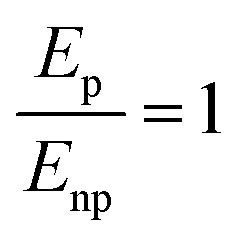
. Determination of the parameters Δ*T*_c_, *δt*, *n*_c_ and *n*_0_ requires caution. Notably, Δ*T*_c_ depends on *R*_np_ due to interface resistance (increases for smaller *R*_np_). Furthermore, *δt*, and *n*_0_ depend on *t*_p_ and can be calculated numerically based on the threshold laser intensity and critical optical breakdown density *n*_c_ for plasma-mediated cavitation. The breakdown time *δt* increases sub-linearly as a function of *t*_p_, while *n*_0_ increases abruptly for *t*_p_ < 0.25 ps, *i.e.*, when multiphoton ionization becomes important. A numerical application is demonstrated in Section S3 of the ESI.† Based on that, the transition curves are shown in Fig. 5b, in fair agreement with the energy ratios shown in Fig. 5a.

### Cavitation threshold criteria

**Fig. 6:**
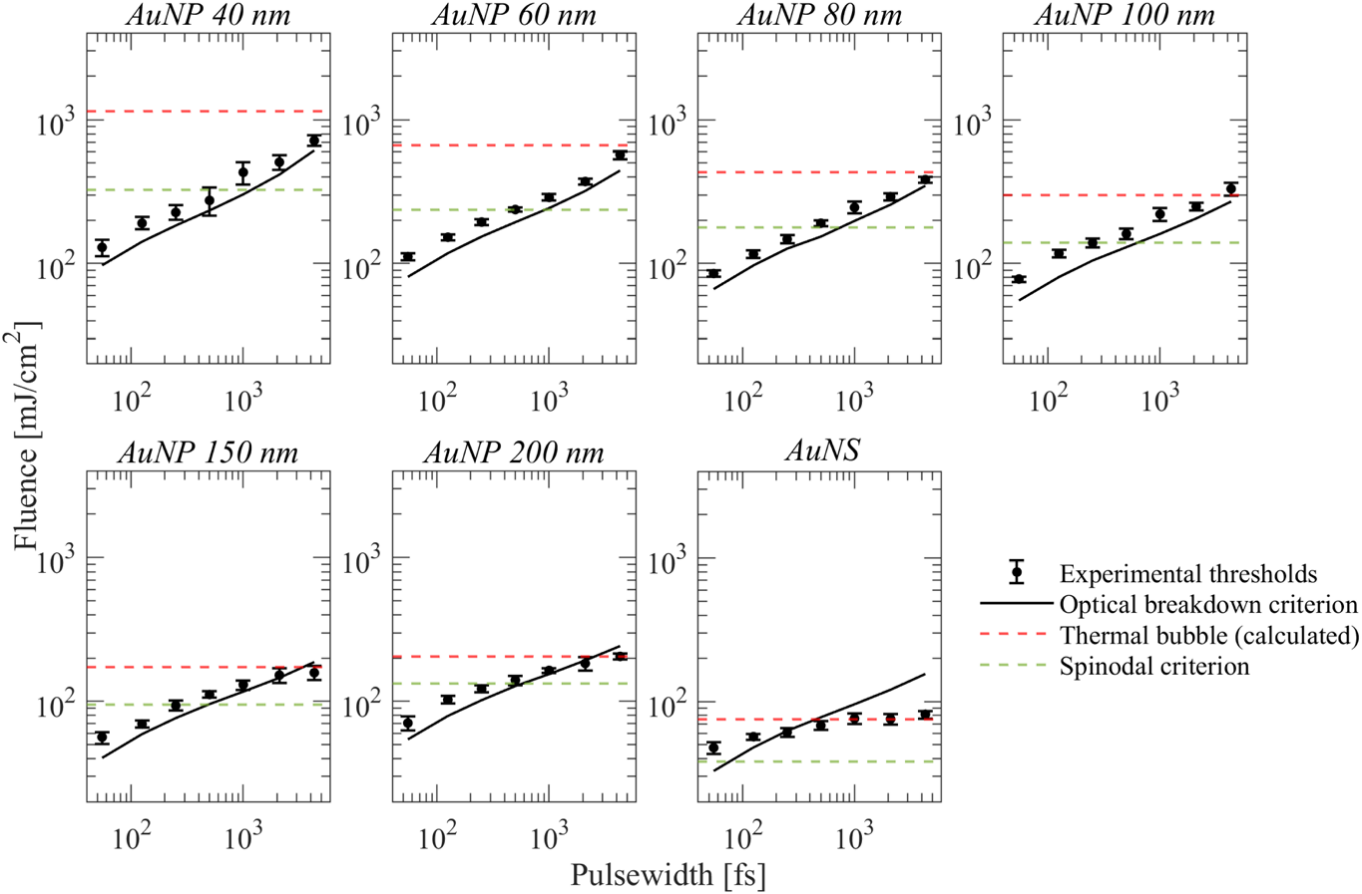
Results of numerical simulations obtained by eqn (S2) (ESI†) and comparison to experimental measurements (black circles).

Generally, at the cavitation thresholds detected experimentally, the maximum calculated densities at the poles (*r* = 0 nm) of the particles are not constant (Fig. 4a). Based on that observation, Dagallier *et al.*^23^ argued that the optical breakdown density of ∼10^21^ cm^−3^ as the bubbling criterion becomes questionable. Even so, for the case of photothermal bubbles, a bubbling criterion has been reported to occur when a thin layer of ∼1–2 nm around a nanoparticle is heated at the spinodal temperature of water.^18^ This indicates that the distance *r* is an important parameter for probing the spinodal water temperature as a sufficient criterion for bubble nucleation, which is related to how wetting conditions affect the distance at which the local density of water reaches its critical density. It is fair to assume then that the same criterion must apply to the case of heating a thin layer of water by plasma relaxation in the case of the plasma-mediated cavitation process. Interestingly, a calculation of the electronic densities at *r* = 1.5 nm away from the particle (Fig. 4a) exhibits a significantly smaller size variation as opposed to the maximum calculated densities (for cases where *E*_p_/*E*_NP_ > 1). An overall pulse width dependence is still observed (calculated maximum attained plasma density is typically higher by 1.5–3 times at the experimental threshold for 55–125 fs excitation compared to 2.0–4.3 ps) in accordance with the plasma dynamics influence described throughout the double pulse experiments. Conclusively, considering the aforementioned limitations, we will approximate the bubbling criterion for the plasma-mediated process by the optical breakdown density of ∼10^−21^ cm^−3^ obtained at 1.5 nm away from the particle surface, independently of the particle size. The calculations are shown in Fig. 6 (optical breakdown criterion, black solid lines).

It will be further useful to examine how the spinodal criterion compares with the thresholds of detectable photothermal bubbles by the employed technique. The latter will be approximated through the experimental results of AuNS for long pulse widths, where photothermal bubbles are most likely excited. Indeed, this is observed as a leveling off on the corresponding fluence threshold for pulse widths >500 fs, which is further corroborated by calculations shown in Fig. 5a where *E*_p_/*E*_NP_ < 0.1. By solving a two-temperature model coupled with a heat diffusion equation at this fluence, we estimated the energy density required for the generation of the detected bubbles around a AuNS (ESI†). The results are shown in Fig. 6 for all other AuNP (thermal bubble (calculated), red dashed lines), along with the corresponding fluence required for water to reach its spinodal at 1.5 nm from the particle surface (spinodal criterion, green dashed lines). The corresponding temperature change for the thermal bubbles (calculated) fluence thresholds are shown in Fig. S3a of the ESI.†

For AuNP of sizes ∼40–100 nm particles, even though the spinodal criterion is exceeded for pulse widths >500 fs, the measured fluence thresholds do not seem to be affected as they remain consistently above the optical breakdown criterion fluence. It is further observed that the calculated required fluence for a detectable photothermal bubble is consistently larger than all experimental threshold fluences, so, presumably, plasma-mediated cavitation dominates the whole range of examined pulse widths. Contrarily, in the case of AuNP of sizes ∼150 and 200 nm, the calculated required fluence for a photothermal bubble is attained for pulse widths >2.0 ps (Fig. 6). Furthermore, the fluence thresholds are nearly equal or smaller than the optical breakdown criterion curve at these pulse widths (*i.e.*, the fluence begins to level off as a function of pulse width), which indicates a transition to photothermal cavitation, much like the case of AuNS bubbles for pulse widths >500 fs. Therefore, the excess energy required for bubble detection at longer pulse-widths (compared to 55 fs) among gold particles is minimum for ∼150 and 200 nm sizes. These observations are consistent with the results shown in Fig. 5, showing that photothermal effects become dominant when *E*_p_/*E*_NP_ < 0.1. Remarkably, the forgoing discussion shows that the transition to photothermal cavitation does not occur abruptly as a function of pulse width, yet an intermediate regime exists where the two mechanisms are comparable. For particles of sizes between 40–100 nm, the excess fluence above the optical breakdown criterion to detect bubbles of ∼0.4–0.5 μm is nearly insensitive to particle size variations in the examined range but increases for longer pulse widths toward the picosecond regime. By contrast, for a photothermal process, the excess fluence above the bubble cavitation criterion varies significantly with particle size. For instance, the spinodal criterion fluence, and the fluence of detectable photothermal bubbles for 40 nm AuNPs differ by more than a factor of 3. Thus, large discrepancies between measurements by the optical scattering technique and calculated values are very likely to occur once a photothermal cavitation process is detected. Finally, we note that, in principle, one needs to account for thermodynamic transitions implicated at such fluencies (see ESI, Section S4†). For practical applications, where ∼μm bubbles or elevated pressures are required, volatile explosion modes must be attained in photothermal processes.^41,45,46^ For plasma-mediated cavitation, on the other hand, stress confinement and ∼μm bubbles are readily achieved due to the ultrafast energy deposition and the highly nonlinear nature of the involved photo-ionization mechanisms.

## Conclusions

Fluence thresholds for ultrafast pulsed (55 fs to 4.3 ps) laser cavitation around various AuNPs (spherical AuNPs, sizes 40–200 nm, and AuNS with SPR peak ∼660 nm) were determined experimentally. The thresholds refer to those of detectable bubbles of 0.4–0.5 μm measured by an optical scattering technique, which was systematically analyzed and tested. Experiments were further performed under double 55 fs pulse excitation of varying pulse delays (0–4.0 ps) to determine the influence of plasma dynamics on the process of *plasma-mediated* cavitation. All experimental results assisted in distinguishing the excitation of either *photothermal* or *plasma-mediated* bubbles. The criterion of reaching the breakdown density at 1–2 nm away from a particle surface as the bubble criterion of *plasma-mediated* cavitation was examined, in analogy to the spinodal criterion of *photothermal* cavitation around plasmonic nanoparticles. Analysis of the experimental data, coupled with calculations based on simple numerical models (electron density rate equation and two-temperature model), led to the following conclusions:

• At a given wavelength, the threshold laser fluence for cavitation in the water around gold nanoparticles is governed by their size, the near field amplification *N*, the electron diffusion length *Λ*, the volumetric absorption *σ*_abs_/*V*_np_ and the laser pulse width. The parameters *N*, *Λ* are related to the induction of plasma adjacent to a nanoparticle, while *σ*_abs_/*V*_np_ to photothermal effects. A relationship between these parameters exists, determining the crossover pulse width *t*_p,t_ of the transition from plasma-mediated to photothermal cavitation process.

• Provided that the cavitation process is plasma-mediated, the calculated maximum (in time) plasma density ∼1.5 nm away from the particle at the threshold of cavitation, is nearly independent of the particle size for a given pulse width. Yet, a small pulse width dependence of the required electron density for plasma-mediated cavitation was estimated (∼3-fold decrease from 55 fs to 4.3 ps). Therefore, the approximative optical breakdown density of ∼10^−21^ cm^−3^ at 1–2 nm away from the particle surface as a bubbling criterion for plasma-mediated cavitation can be applied with caution at a given pulse width.

• The difference between the spinodal criterion fluence and the threshold fluence for detectable photothermal bubbles of size ∼0.4–0.5 μm increases substantially with decreasing particle size. Such observation has important implications for practical applications of photothermal bubbles around nanoparticles. Contrarily, in plasma-mediated nano-cavitation, only weak size dependence of the required excess fluence above the optical breakdown criterion was noted.

## Conflicts of interest

There are no conflicts to declare.

## Supplementary Material

NA-005-D3NA00743J-s001
