# Supplementary material for: Influence of photothermal and plasma-mediated nano-processes on fluence thresholds for ultrafast laser-induced cavitation around gold nanoparticles
Source: Nanoscale Adv. 2023 Oct 20;5(24):6887–96. doi: 10.1039/d3na00743j (PMC10696957; doi:10.1039/d3na00743j)
Supplement: NA-005-D3NA00743J-s001 [file NA-005-D3NA00743J-s001.pdf]

Electronic Supplementary Material (ESI) for Nanoscale Advances.
This journal is © The Royal Society of Chemistry 2023

# Supplementary Information: Influence of photothermal and plasma-mediated nanoprocesses on fluence thresholds for ultrafast laser-induced cavitation around gold nanoparticles

Leonidas Agiotis,[a] Vi Tching De Lille[a] and Michel Meunier*[a]

[a.] *Department of Engineering Physics, Polytechnique Montréal, Montreal, QC H3C 3A7, Canada*
**E-mail: michel.meunier@polymtl.ca*

## S1. Optical Setup characterization

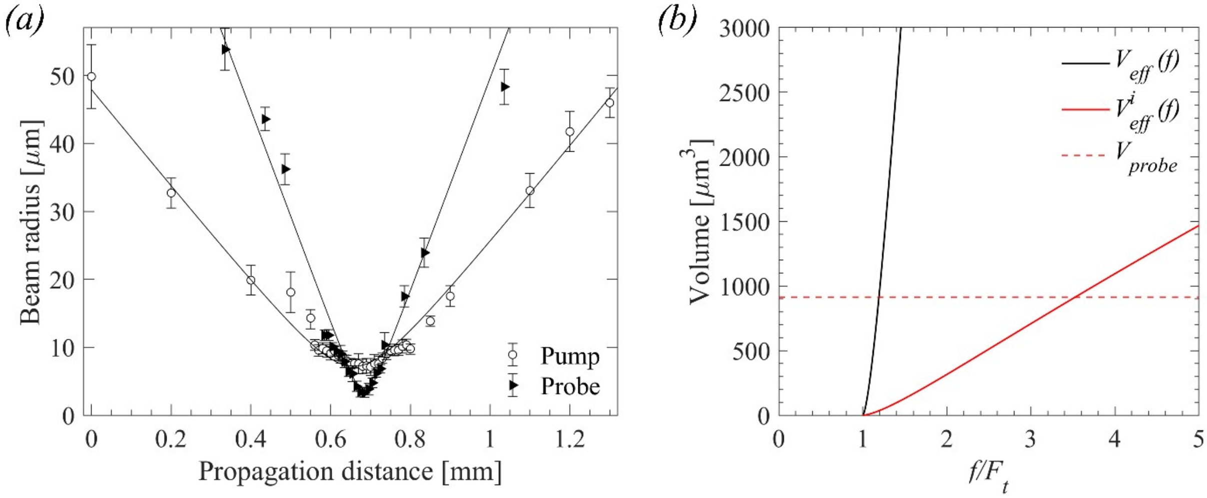


**Figure S1.** (a) Pump and probe laser 1/e$^2$ beam radius characterization as a function of propagation distance z. The probe beam is focused in a considerably smaller volume. (b) the effective volume as a function of the normalized fluence $F$. The solid black curve shows the excitation volume and the red solid volume imaged by the probe's effective volume. For reference, the volume at the field of focus of the probe is shown by a dashed red line.

## S2. Numerical simulations

### a) Electron density rate model (plasma-mediated cavitation)

Free electron density $n$ at a specified location near the poles of a nanoparticle (i.e., where field amplification occurs) following excitation, can be estimated by a well-known rate equation [1-5] further modified to consider thermionic emission from the particle:

$$\frac{\partial n}{\partial t} + \nabla \cdot j^n = S_g + S_p + S_c n - S_r n^2 \qquad \text{(S1)}$$

where $j^n$ is the electron flux due to diffusion, $S_g$ is the thermionic emission rate, $S_p$ stands for the photoionization rate, $S_c$ denotes the collision ionization rate and $S_r$ is the recombination rate. The gradient of the electron density flux is given by the expression $\nabla \cdot j^n \approx D_e n/\Lambda^2$, where $D_e = \tau \mathrm{E}_{av}/(3m_e)$ is the diffusion coefficient, $\mathrm{E}_{av} = 5\tilde{\Delta}/4$ is the

average energy of electrons, $\tau$ is the time between collisions, $\tilde{\Delta} = \frac{2}{\pi}\Delta\sqrt{\frac{\gamma^2}{1+\gamma^2}}$ is the effective ionization potential, $\Delta$ is the ionization potential of water, $\gamma = \frac{\omega}{e}\sqrt{\frac{m' c\varepsilon_0 n_0 \Delta}{2N^2 I}}$ is the Keldysh parameter, $N$ is the plasmonic near-field amplification, $I$ is the laser peak intensity, ω is the laser radial frequency, $m_e$ is the electron mass, $e$ is the elementary charge, $c$ is the speed of light, $\varepsilon_0$ is vacuum permittivity, $n_0$ is the refractive index of the medium and $\Lambda$ is a characteristic diffusion length. The latter is estimated by the extension of near-field around a nanoparticle (Table 1 of main text). The rate $S_p$, is calculated by the expression $S_p = (2/9\pi)\omega\left(m'\omega/\hbar\right)^{3/2} exp(2\kappa)\Phi\left(\sqrt{2k - 2\tilde{\Delta}/\hbar\omega}\right)\left[e^2N^2I/\left(8m'\tilde{\Delta}\omega^2 c\varepsilon_0 n_0\right)\right]^{\kappa}$, where $m' = 0.64m_e$ the reduced electron mass, $\hbar$ is the reduced Planck's constant, $\kappa =$ is the number of simultaneously absorbed photons to cross $\tilde{\Delta}$, and $\Phi$ is the Dawson function. The rate $S_c$ is calculated by the expression $S_c = H\left(n\Lambda^3 - \frac{1}{2}\right)\frac{\eta}{1 + \eta\tau\kappa}$, where $H()$ is the Heaviside step function, $\eta = \frac{1}{\omega^2\tau^2 + 1}\left(\frac{e^2\tau N^2 I}{cn_0\varepsilon_0 m_e(3\tilde{\Delta}/2)} - \frac{m_e\omega^2\tau}{M}\right)$ and $M$ is the mass of the water molecule. The plasmonic near-field amplification $N$ was estimated based on Mie theory as a function of distance from the particle surface [6] (Table 1 of main text). The rate of thermionic emission has been calculated by the relation [7, 8] $S_g = \frac{1}{e}\frac{\partial}{\partial r}J_{r=0} \approx \frac{1}{e}\frac{A_{int}}{V_{np}}J_{r=0}$, where $A_{int}$ denotes the intersection surface between the particle and water, $V_{np}$ is the volume of the nanoparticle and the current density at the interface $J_{r=0}$ was calculated based on the generalized Fowler-DuBridge theory for nonlinear photoemission (considering one and two-photon absorption) [9], as $J_{r=0} = \sum_{n=1}^{2} a_n\left(\frac{e}{\hbar\omega}\right)^2 T_e^2 A_0 F\left(\frac{n\hbar\omega - \varphi}{k_B T_e}\right) I^n + A_0 T_e^2 exp\left(-\frac{\varphi}{k_B T_e}\right)$, where $a_n$ are $n$ photon absorption parameters for Au, $\varphi$ is the electron extraction potential, $T_e$ is the temperature of the electrons of the particle, $k_B$ is the Boltzmann constant and $A_0$ is the Richardson coefficient and $F$ stands for the Fowler function. The temperature $T_e$ is calculated by coupling equation (S1) to a two-temperature model shown below, i.e., equations (S2). Of note, the contribution by thermionic emission to plasma generation around a given nanoparticle was negligible in comparison to contribution from nonlinear absorption in water, at all experimentally determined thresholds of cavitation for all spherical Au nanoparticles.

**Table S1.** Parameters used for the numerical simulations.

| Parameter | Symbol | Value | Ref. |
|---|---|---|---|
| | | **Rate equation** | |
| Free time between collisions | $\tau$ | $= 1.7\,fs$ | [5] |
| Water ionization potential | $\Delta$ | $= 6.5\,eV$ | [10] |
| Water refractive index | $n_0$ | = 1.33 | |
| Water molecular mass | $M$ | $= 3 \times 10^{-26}\,kg$ | [11] |
| Number of photons required for multiphoton ionization (800 nm) | $\kappa$ | $= 5$ | |
| Recombination rate | $S_r$ | $= 3 \times 10^{-15}\,m^3 s^{-1}$ | [12] |
| One-photon thermionic emission parameter | $a_1$ | $= 1 \times 10^{-14}\frac{cm^2}{A}$ | [9] |
| Two-photon thermionic emission parameter | $a_2$ | $= 4 \times 10^{-23}\frac{cm^4}{A^2}$ | [9] |

| | | | |
|---|---|---|---|
| Richardson coefficient | $A_0$ | $= 120 \frac{A}{cm^2 K^2}$ | [7] |
| Electron extraction potential | φ | $= 3.72\ eV$ | [8] |
| **Two temperature model** | | | |
| Au density | $\rho_{Au}$ | $= 19300\ kg\ m^{-3}$ | |
| Electron-phonon coupling parameter | $G$ | $= 2.5 \times 10^{16}\ W\ m^{-3}\ K^{-1}$ | [13] |
| Lattice heat capacity | $C_l$ | $= \rho_{Au}\left(119 + 3.061 \times 10^{-2} T_l\right)\ (J\ m^{-3}\ K^{-1})$ | [14, 15] |
| Electron heat capacity | $C_e$ | $= 70 T_e\ J\ m^{-3}\ K^{-1}$ | [16] |
| Water thermal conductivity | $k_w$ | $= 0.58\ W\ m^{-1}\ K^{-1}$ | |

b) Two-temperature model (photothermal cavitation)

The two-temperature model used to estimate the temperature rise of the gold lattice and adjacent water under photo-excitation reads [1, 14, 15, 17]:

$$
\begin{aligned}
C_e(T_e)\frac{dT_e}{dt} &= -G(T_e - T_l) + \frac{\sigma_{abs} I(t)}{V_{np}} \\
C_l(T_l)\frac{dT_l}{dt} &= G(T_e - T_l) - \frac{g}{R_{np}}\left(T_l - T_w\big|_{r = R_{np}}\right) \\
C_w(T_w)\frac{\partial T_w}{\partial t} &= \frac{1}{r^2}\frac{\partial}{\partial r}\left(k_w r^2 \frac{\partial T_w}{\partial r}\right)
\end{aligned}
\tag{S2}
$$

where the subscripts $e$, $l$ and $w$ stand for "electron", "lattice" and "water", respectively, $C$ denotes the heat capacity, $G$ is the electron-phonon coupling, $g = 3\frac{C_w k_w}{R_{NP} C_l}$ is the interface (Au-water) conductance, $R_{np}$ is the radius of the Au particle, $k_w$ the thermal conductivity of water and $I(t)$ the laser pulse intensity, formulated by $I(t) = 2\frac{F}{t_p}\sqrt{\frac{ln2}{\pi}}e^{-4ln2\frac{t^2}{t_p^2}}$ where $F$ is the laser pulse fluence and $t_p$ stands for the FWHM (full width at half maximum) pulse width.

Following Dagallier et al. [1, 17] the energy $E_{NP \to w}$ transferred from the AuNS to surrounding water at a fluence ~80 mJ/cm$^2$ (2.1-4.3 ps pulse range) was estimated by time integration of the heat fluence at the surface of the particle over the characteristic time of phonon-phonon thermalization $\tau_g = \rho_{Au} C_l / (3 g R_{NP})$ ($\rho_{Au}$ denotes Au density) at the interface of the two materials, i.e., by $E_{NP \to w} \approx -\int_0^{\tau_g} 4\pi R_{NP}^2 g(T_w - T_l)dt$. The energy density $U$ deposited within a thin 1.5 nm layer volume $V_c$ around the AuNS is calculated by the ratio $U = E_{NP \to w}/V_c$. We have subsequently estimated the required fluence of a 4.3 ps pulse to reach the same energy density deposition $U$ for all other spherical AuNPs, which we denoted as the threshold fluence of detectable thermal bubbles.

c) Experimental fluence thresholds at a pulsewidth of 55 fs and comparison to theoretical calculations

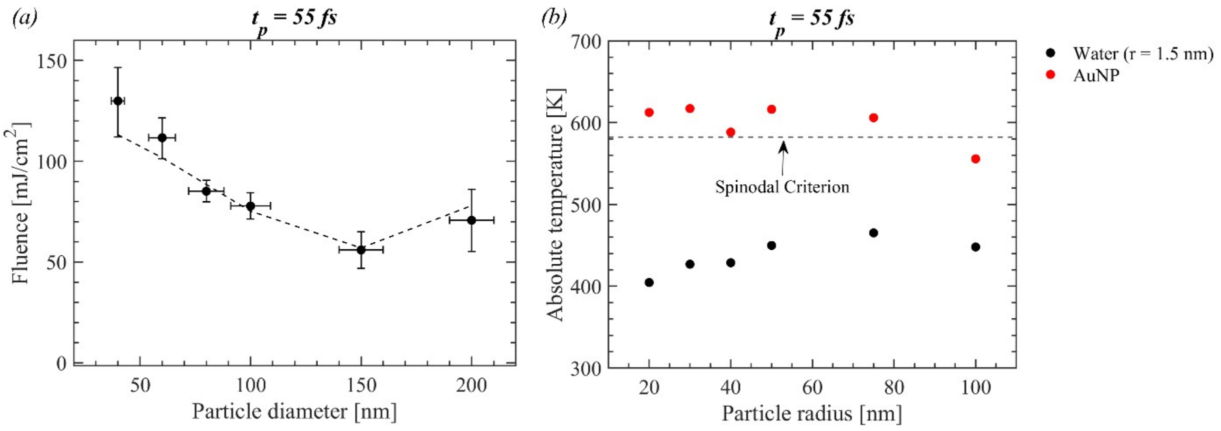


**Figure S2.** (a) Experimental fluence thresholds at a pulsewidth of 55 fs (black circles). The black dashed-line corresponds to the calculation $F_{th}(R_{np}) = F_{th,min} \times N_{max}{}^2/N^2(R_{np})$, where $R_{np}$ is the radius of the AuNP, $F_{th,min} = F_{th}(R_{np} = 75\ nm)$ and $N_{max} = N(R_{np} = 75\ nm)$. The experimental measurements are in fair agreement with the calculation, implying a plasma-mediated cavitation process. (b) This is further supported by the calculated absolute water temperature (maximum in time and at a distance *r* = 1.5 nm from the particle surface) at the fluence thresholds. It is seen that water temperature is consistently lower than the 90% of the water spinodal (~580 K), i.e., lower than the spinodal criterion for photothermal cavitation, for all examined particles.

## S3. Relative contributions of photothermal and plasma-mediated processes

In Figure 5a of the main text, a crossover pulse width $t_{p,t}$ for the transition from plasma-mediated to photothermal nucleation is noted when $E_p/E_{NP} \approx 1$. According to data shown in Figure 5a, that occurs at different pulse widths depending on the examined nanoparticle. Approximately, for AuNS it occurs at ~125 fs ($E_p/E_{NP} \approx 0.74$), for 200 nm AuNP at ~250 fs ($E_p/E_{NP} \approx 1.10$), for 150 nm AuNP at ~500 fs ($E_p/E_{NP} \approx 0.99$), for 100 nm AuNP at ~4.3 ps ($E_p/E_{NP} \approx 1.24$) and for 80 nm AuNP at ~4.3 ps ($E_p/E_{NP} \approx 1.20$). Based on the corresponding laser intensities and pulse widths, we can use equations (S2) to estimate the temperature increase at these conditions for each nanoparticle, which must define $\Delta T_c$. The obtained estimated values are shown in Figure S3a. Unsurprisingly, there is variation in obtained $\Delta T_c$ depending on the particle size. That is mainly because of the interface resistivity at the boundary and the dynamic energy transfer between the gold lattice and water. Generally, it is expected that the temperature increase follows the form:

$$\Delta T_c = {}^{l}/_{R_{np}} \tag{S3}$$

The equation S3 was fit on the data shown in Figure S3a with $l = 4.46 \times 10^{-5}\ [m][K]$.

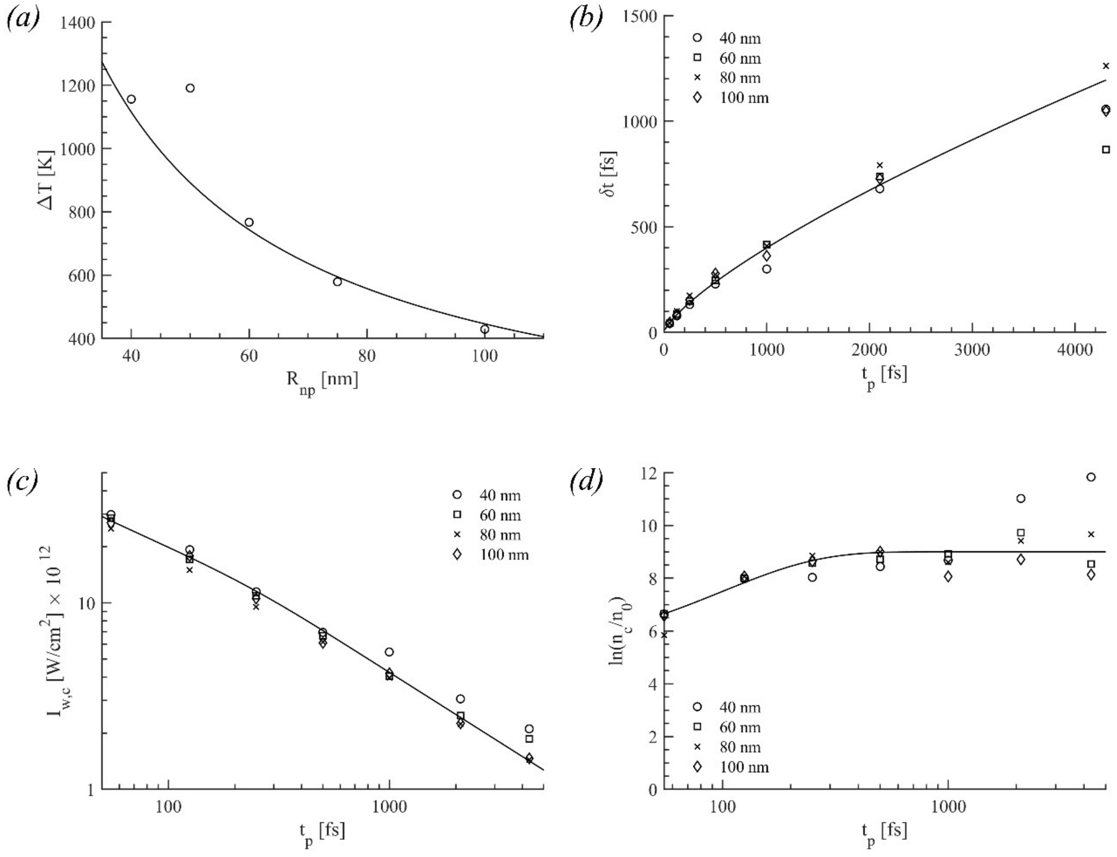


**Figure S3.** (a) The critical temperature $\Delta T_c$ (circles) obtained by solution of equations S2 at the crossover pulse widths determined based on data from Figure 5 of the main text. The fitting equation S3 is shown over the data (solid curve). (b) The breakdown time was calculated by the solution of equation S1 versus pulse width at the experimental thresholds of cavitation of 40-100 nm AuNPs. Equation S2 was fit over the calculated data. (c) The calculated laser intensity $I_{w,c} = N^2 I_t$ for obtaining breakdown-induced bubbles around a particle, where $I_t$ denotes the experimentally obtained threshold intensity of detected bubbles. The solid line is deduced based on the fitting curves of (b) and (d). (d) The electron density growth parameter $ln(n_c/n_0)$ as a function of the laser pulse width. The empirical relation S5 (solid line) is shown against the calculated data.

In the discussion section *"Relative contributions of photothermal and plasma-mediated processes"* of the main text, the laser intensity $I_{w,c}$ for obtaining breakdown accompanied with induction of a nanobubble was evoked. The latter is related to the laser fluence threshold when a plasma-mediated process is involved and to the near-field amplification of each particle. The measured fluencies for the cases were plasma-mediated process dominates cavitation, i.e., for AuNP of sizes 40-100 nm can be used as input parameters to solve equation (S1) and to determine the breakdown time $\delta t$. The obtained data are shown in Figure S3b, exhibiting generally a sublinear relation with the laser pulse width. The behavior is physically interpreted based on the transition to multiphoton-assisted cascade breakdown for pulse widths longer than 200 fs [2]. An empirical sublinear relation is fit between $\delta t$ and $t_p$ that reads:

$$\delta t = C t_p^{4/5} \tag{S4}$$

with $C = 0.0014$.

Next, we can further obtain the local intensities $I_{w,c}$ at the poles of the particles of sizes 40-100 nm, at the experimental thresholds by the relation $I_{w,c} = N^2 I_t$, where $I_t$ stands for the measured input laser intensities at the threshold of

cavitation (shown in Figure S3c). Combining those with relation (S4), the electron density growth parameter $ln\left(n_c/n_0\right)$ can be deduced through the relation $I_{w,c} \approx \frac{mcn_0\varepsilon_0(3\bar{\Delta}/2)(\omega^2\tau^2+1)}{e^2\tau}\frac{ln\left(n_c/n_0\right)}{\delta t}$. Notably, the density $n_0$ includes not only the seed density of the electrons provided by multiphoton ionization but also losses attributed to diffusion and recombination. Therefore, as defined herein, the growth parameter $ln\left(n_c/n_0\right)$ denotes the plasma growth due to cascade ionization, from a seed electron density and against all considered loss mechanisms, to a breakdown density $n_c$. The estimated growth parameter was plotted as a function of $t_p$ for the particles of sizes 40-100 nm in Figure S3d. The empirical relation:

$$ln\left(n_c/n_0\right) = 9 - 1.5e^{1-\frac{t_p}{t_m}} \quad \text{(S5)}$$

was used to fit the data with $t_m = 100$ fs (denoting the transition from multiphoton-dominated to multiphoton-assisted cascade breakdown [2]) with a maximum relative error of <25% for $t_p > 1$ ps for 40 nm particles, and <12% for all particles when $t_p \leq 1$ ps. Plugging equations (S3), (S4), (S5) in equation (4) we can obtain a diagram of the ratio $\frac{\sigma_{abs}\Lambda}{V_{np}N^2}$ versus the crossover pulse width $t_{p,t}$ for the various $R_{np}$ of nanoparticles used experimentally, as shown in Figure S4. The diagram exhibits a fair agreement with Figure 5a of the main text on the crossover pulse width condition (where $\frac{E_p}{E_{np}} = 1$), considering the various approximations made.

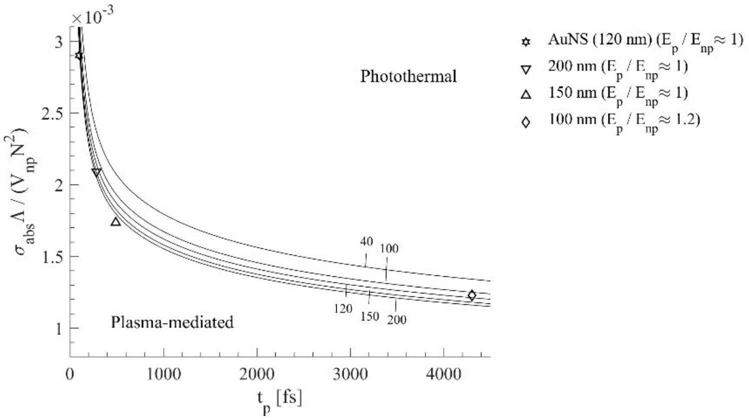


**Figure S4.** Diagram of the ratio $\frac{\sigma_{abs}\Lambda}{V_{np}N^2}$ versus pulse width $t_p$. The solid black curves correspond to the crossover pulse width $t_{p,t}$ that marks the transition from plasma-mediated to photothermal cavitation for various nanoparticle sizes (shown in [nm]). The crossover pulse widths for the cases of AuNS, 200 nm, and 150 nm (based on Figure 5a of the main text, defined as the pulse width where $E_p/E_{NP} \approx 1$), are demonstrated. The case of 100 nm particle, where $E_p/E_{NP} = 1.2$ is also shown for comparison.

Of note, the above analysis assumes that $I_{w,c}$ is size-independent. For instance, in the case of 40 nm particles, the above numerical application neglects the growth of $ln\left(n_c/n_0\right)$ as pulse width becomes longer, which is physically related to the smaller diffusion length, affecting the Debye screening compared to larger particles. Therefore, it is expected that the $\frac{\sigma_{abs}\Lambda}{V_{np}N^2}$ curve is reducing for longer pulse widths and for 40 nm particles, and therefore, is

overestimated in Figure S4. The same applies to any particle for pulse widths $\gtrsim$ 20 ps, where cascade ionization dominates optical breakdown [2]. This is because the growth parameter $ln\left(n_c/n_0\right)$ becomes progressively larger and the breakdown intensity almost constant at a wide range of pulse widths up to the nanosecond regime (see for example Figure 4 of reference [5]). By contrast the slope of the curve of laser intensity threshold for photothermal cavitation versus pulse width does not change, since it depends entirely on the deposited energy. So, expectedly, the curve $\frac{\sigma_{abs}\,\Lambda}{V_{np}\,N^2}(t_p = t_{p,t}) \rightarrow 0$ (i.e., $N^2$ must take very large values for a plasma-mediated transition) for pulse widths $\gtrsim$ 20 ps and cavitation is eventually governed entirely by photothermal processes in the ns pulsed regime.

## S4. Thermodynamic transitions of the AuNP lattice and accompanied effects

We have calculated (based on equations (S2)) the required laser fluence that results in non-reversible heating, i.e., fluencies that result in the heating of gold beyond its melting point of ~1330 K [18]. The results show that this occurs for the following cases: for 40 nm, 60 nm, 80 nm, and 100 nm particles at a pulse width of 4.3 ps and for 40 nm particles at a pulse width of 2 ps, which indicates the possibility of having shape relaxation under these conditions. Size reduction would be possible via Coulomb explosion if electronic temperatures exceed about 7300-8000 K [15], or the boiling point of Au is exceeded. Even though the latter never occurs at all observed thresholds, electronic temperatures exceed 7300 K for spherical particles of 40 nm, 60 nm, and 80 nm, for pulse widths > 1 ps, and for 100 nm particles for pulse widths > 2ps, which indicates the possibility of size reduction for those particles. The above conditions occur already below the threshold for detection of purely photothermal bubbles of diameter ~0.4-0.5 μm. Thus, this observation shows that there are important implications for practical applications (requiring bubbles of > 0.5 μm diameter) because of the significant discrepancy between the very threshold for explosive cavitation (spinodal criterion) and the threshold for detectable photothermal bubbles of diameter ~0.4-0.5 μm. As has been discussed in the past, explosive modes must be attained for nano-photo-thermolysis of cancer when it comes to laser-induced photothermal bubble excitation around gold nanoparticles [19].

## References


[1] É. Boulais, R. Lachaine, M. Meunier, Plasma mediated off-resonance plasmonic enhanced ultrafast laser-induced nanocavitation, Nano letters 12(9) (2012) 4763-4769.

[2] P.K. Kennedy, D.X. Hammer, B.A. Rockwell, Laser-induced breakdown in aqueous media, Progress in quantum electronics 21(3) (1997) 155-248.

[3] R.m. Lachaine, E.t. Boulais, M. Meunier, From thermo-to plasma-mediated ultrafast laser-induced plasmonic nanobubbles, Acs Photonics 1(4) (2014) 331-336.

[4] J. Noack, A. Vogel, Laser-induced plasma formation in water at nanosecond to femtosecond time scales: calculation of thresholds, absorption coefficients, and energy density, IEEE journal of quantum electronics 35(8) (1999) 1156-1167.

[5] A. Vogel, J. Noack, G. Hüttman, G. Paltauf, Mechanisms of femtosecond laser nanosurgery of cells and tissues, Applied physics B 81 (2005) 1015-1047.

[6] R. Lachaine, E. Boulais, D. Rioux, C. Boutopoulos, M. Meunier, Computational design of durable spherical nanoparticles with optimal material, shape, and size for ultrafast plasmon-enhanced nanocavitation, ACS Photonics 3(11) (2016) 2158-2169.

[7] N. Bulgakova, R. Stoian, A. Rosenfeld, I. Hertel, E. Campbell, Electronic transport and consequences for material removal in ultrafast pulsed laser ablation of materials, Physical Review B 69(5) (2004) 054102.

[8] T. Labouret, B. Palpant, Nonthermal model for ultrafast laser-induced plasma generation around a plasmonic nanorod, Physical Review B 94(24) (2016) 245426.

[9] J. Girardeau-Montaut, C. Girardeau-Montaut, Theory of ultrashort nonlinear multiphoton photoelectric emission from metals, Physical Review B 51(19) (1995) 13560.

[10] F. Williams, S. Varma, S. Hillenius, Liquid water as a lone-pair amorphous semiconductor, The Journal of Chemical Physics 64(4) (1976) 1549-1554.

[11] P.K. Kennedy, A first-order model for computation of laser-induced breakdown thresholds in ocular and aqueous media. I. Theory, IEEE journal of quantum electronics 31(12) (1995) 2241-2249.

[12] F. Docchio, Lifetimes of plasmas induced in liquids and ocular media by single Nd: YAG laser pulses of different duration, Europhysics Letters 6(5) (1988) 407.

[13] P. Grua, J. Morreeuw, H. Bercegol, G. Jonusauskas, F. Vallée, Electron kinetics and emission for metal nanoparticles exposed to intense laser pulses, Physical Review B 68(3) (2003) 035424.

[14] M. Strasser, K. Setoura, U. Langbein, S. Hashimoto, Computational modeling of pulsed laser-induced heating and evaporation of gold nanoparticles, The Journal of Physical Chemistry C 118(44) (2014) 25748-25755.

[15] D. Werner, S. Hashimoto, Improved working model for interpreting the excitation wavelength-and fluence-dependent response in pulsed laser-induced size reduction of aqueous gold nanoparticles, The Journal of Physical Chemistry C 115(12) (2011) 5063-5072.

[16] J. Hohlfeld, S.-S. Wellershoff, J. Güdde, U. Conrad, V. Jähnke, E. Matthias, Electron and lattice dynamics following optical excitation of metals, Chemical Physics 251(1-3) (2000) 237-258.

[17] A. Dagallier, E. Boulais, C. Boutopoulos, R. Lachaine, M. Meunier, Multiscale modeling of plasmonic enhanced energy transfer and cavitation around laser-excited nanoparticles, Nanoscale 9(9) (2017) 3023-3032.

[18] K. Koga, T. Ikeshoji, K.-i. Sugawara, Size-and temperature-dependent structural transitions in gold nanoparticles, Physical review letters 92(11) (2004) 115507.

[19] R.R. Letfullin, C. Joenathan, T.F. George, V.P. Zharov, Laser-induced explosion of gold nanoparticles: potential role for nanophotothermolysis of cancer, (2006).
